# Lurasidone for Treating Schizophrenia and Bipolar Depression: A Review of Its Efficacy

**DOI:** 10.7759/cureus.38071

**Published:** 2023-04-24

**Authors:** Martin Tarzian, Majd Soudan, Muhammed Alhajji, Mariana Ndrio, Adegbenro O Fakoya

**Affiliations:** 1 Psychiatry, University of Medicine and Health Sciences, Basseterre, KNA; 2 Psychiatry, Nuvance Health Medical Practice - Primary Care Carmel, New York, USA; 3 Internal Medicine, Augusta University/University of Georgia Medical Partnership, Athens, USA; 4 Cellular Biology and Anatomy, Louisiana State University Health Sciences Center, Shreveport, USA

**Keywords:** clinical global impressions scale for use in bipolar illness, children's depression rating scale-revised, montgomery-asberg depression rating scale, clinical global impression-severity scale, positive and negative syndrome scale, brief psychiatric rating scale, bipolar depression, schizophrenia, lurasidone

## Abstract

Lurasidone is an antipsychotic medication that blocks dopamine D2 and serotonin 5-hydroxy-tryptamine (5-HT)_2A _receptors and affects other serotoninergic and noradrenergic receptors. It has rapid absorption and linear pharmacokinetics. The rates of metabolic syndrome for patients taking lurasidone are comparable to placebo groups. Lurasidone is a safe and effective treatment for patients with acute schizophrenia and bipolar depression. It has been found to improve the brief psychiatric rating scale and other secondary measures in schizophrenic patients and reduce depressive symptoms in bipolar I depression. The once-daily administration of lurasidone is generally well-tolerated and does not cause clinically significant differences in extrapyramidal symptoms, adverse effects, or weight gain compared to a placebo. However, lurasidone's effectiveness in combination with lithium or valproate has been mixed. Further research is needed to determine optimal dosing, treatment duration, and combination with other mood stabilizers. Long-term safety and effectiveness and its use in different subpopulations should also be evaluated.

## Introduction and background

Schizophrenia and bipolar depression are serious mental illnesses that can significantly impact individuals, families, and society. Approximately 1% of the general population is diagnosed with schizophrenia [1], while 1.8% have bipolar disorder [2]. For individuals with schizophrenia and bipolar depression, their quality of life is drastically decreased; therefore, effective treatment is essential to help manage these symptoms and improve their well-being. Lurasidone, a second-generation atypical antipsychotic slowly metabolizing medication, has been proven to be successful in treating both disorders [1,2]. Recent literature demonstrated outstanding safety and favorable metabolic adverse effect profiles, making it a compelling option in treating schizophrenia and bipolar disorder [3]. Lurasidone treatment is associated with significantly less weight gain when compared to olanzapine and quetiapine [4,5]. Although there was no significant difference in the adverse effects compared to ziprasidone, lurasidone showed lower rates of somnolence [6]. The sedating effect of lurasidone, although similar to other antipsychotic medications is, however, of a lesser extent when compared to most agents [7,8]. Lurasidone, like risperidone, has a demonstrable dose-dependent increase in prolactin levels [9] although to a lesser extent [10].

Furthermore, it has improved the quality of life in individuals suffering from schizophrenia and bipolar disorder by improving psychotic and depressive symptoms in both populations. This review delves into the potential of lurasidone, an antipsychotic medication, in treating schizophrenia and bipolar depression. This paper examines how lurasidone fits the larger treatment landscape for these mental health disorders.

## Review

Pharmacological profile

Chemically, lurasidone is very similar to perospirone and ziprasidone. It belongs to the chemical group benzisothiazoles. Other members of this chemical class include the benzisoxazole derivatives risperidone, paliperidone, and iloperidone [1]. Lurasidone, like other currently available antipsychotic agents, can block dopamine D2 receptors [3]. Additionally, it shares the characteristic of second-generation antipsychotics for blocking the serotonin 5-hydroxy-tryptamine (5-HT) 2A receptor [3]. Furthermore, lurasidone is a potent antagonist at the serotonin 5-HT7 receptor, a moderately potent partial agonist at the serotonin 5-HT1A receptor, and a moderately potent antagonist at the noradrenergic alpha2C receptors. It has little affinity for the serotonin 5-HT2C, histamine H1, muscarinic cholinergic M1, or noradrenergic alpha1 receptors [3].

Lurasidone is quickly absorbed and reaches its highest plasma concentration levels within 1.5 to three hours [11,12]. The pharmacokinetics of lurasidone is linear for the dose range of 20 mg to 160 mg. The body reaches a steady state with lurasidone within seven days [11-13]. For a dose of 40 mg, the volume of distribution is estimated to be 6173 liters, and the clearance is reported to be 3902 mL/min [13]. The elimination half-life in healthy subjects in single-dose pharmacokinetic studies (doses less than 100 mg/day) yielded a mean terminal half-life ranging from 12.2 to 18.3 hours but rose to between 28.8 and 37.4 hours at a steady state in individuals with schizophrenia. However, lurasidone plasma steady-state concentration levels were reached within seven days in individuals with schizophrenia [1]. 

Lurasidone binds strongly to plasma proteins, specifically albumin, and α1-glycoprotein, and is primarily metabolized by the liver enzyme cytochrome P450 3A4 (CYP3A4) into three active and two inactive metabolites [1]. The primary active metabolite, ID-14283, is rapidly present in the serum with a maximum concentration equal to 26% of the initial lurasidone dose and has a similar pharmacological profile to lurasidone but a shorter life span of 7.48 to 10 hours. The other two metabolites, ID-14326 and ID-11614, are present at shallow levels of 3% and 1%, respectively [3,11].

Lurasidone crosses the placental barrier and is excreted in urine and stools, with approximately 89% found in these excrements. After administering lurasidone, 80% of the radioactivity was found in stools and 9% in urine [11,12]. Maximum concentration and area under the curve values increased in patients with mild, moderate, or severe renal and hepatic insufficiency. This indicates that dosages may need to be adjusted for these patients [11,14]. There does not seem to be an impact of race or age on the pharmacokinetics of lurasidone. Blood tests performed on elderly psychotic patients who took 20 mg/day of lurasidone showed concentrations similar to those found in younger subjects [1,15].

Metabolic profile

A recent analysis by Tocco et al. showed metabolic syndrome rates during treatment with lurasidone (ranging from 40 to 160 mg/day) [16]. This analysis was based on pooled, short-term data from three randomized, double-blind, placebo-controlled trials comparing lurasidone to olanzapine and quetiapine extended-release (XR). Moreover, the research incorporated long-term data from two active-comparator-controlled studies comparing lurasidone to risperidone and quetiapine XR and data from two open-label studies where patients were switched from olanzapine or risperidone to lurasidone [16].

Results demonstrated that the likelihood of meeting the criteria for metabolic syndrome at week six was comparable between the lurasidone and placebo groups. However, the odds of meeting metabolic syndrome criteria were significantly higher for patients receiving olanzapine and quetiapine than placebo [16]. No variation in the likelihood of metabolic syndrome was observed across the lurasidone dose range of 40 to 160 mg/day. The long-term studies revealed that the odds of having metabolic syndrome after 12 months of treatment were significantly higher for patients receiving risperidone and quetiapine XR than those receiving lurasidone [16]. In the open-label extension studies, the rate of metabolic syndrome decreased among patients who switched to lurasidone after six weeks of olanzapine treatment or 12 months of risperidone treatment [16]. This analysis of clinical trials on lurasidone found that the risk of developing metabolic syndrome was low when taking the drug for short- and long-term periods at 40 to 160 mg per day [16].

Lurasidone in treating schizophrenia

Acute Exacerbation of Schizophrenia

In a study by Nakamura et al., 90 patients were randomly assigned to receive six weeks of lurasidone 80 mg or placebo as part of a double-blind treatment [17]. The primary measure of efficacy was the brief psychiatric rating scale (BPRS). At the end of the study, treatment with lurasidone significantly improved the BPRS score compared to placebo [17]. This improvement was also seen in all secondary measures, including the positive and negative syndrome scale (PANSS) total score and the PANSS positive, negative, and general psychopathology subscales [17]. In the study, lurasidone treatment was generally well-tolerated. It did not have any adverse effects on metabolic or electrocardiogram parameters, nor did it cause clinically significant differences in extrapyramidal symptoms compared to placebo. The study's results indicated that lurasidone is a safe and effective treatment for patients with an acute exacerbation of schizophrenia [17].

Loebel et al. found similar results to Nakamura et al. in their trial [18]. Patients with an acute exacerbation of schizophrenia who were recently admitted inpatients were assigned randomly to six weeks of once-daily, fixed-dose, double-blind treatment with either lurasidone 80 mg, lurasidone 160 mg, quetiapine XR 600 mg, or placebo. The efficacy was measured by analyzing the change from baseline to week six in the PANSS and clinical global impression-severity scale (CGI-S) score [18]. Both doses of lurasidone and quetiapine XR 600 mg showed significant improvement in PANSS total score, PANSS positive and negative subscale scores, and CGI-S score compared to placebo at week six. The endpoint responder rate was higher for those treated with lurasidone 80 mg, lurasidone 160 mg, and quetiapine XR 600 mg than placebo [18]. The proportion of patients experiencing at least 7% weight gain was 4% for each lurasidone group, 15% for the quetiapine XR 600 mg group, and 3% for the placebo group [18]. The endpoint changes in cholesterol, triglycerides, and low-density lipoprotein (LDL) cholesterol levels were similar between both lurasidone groups and placebo. In contrast, the quetiapine XR 600 mg group showed a significant increase in cholesterol, LDL cholesterol, and triglycerides compared to the placebo [18].

Long-term Treatment for Schizophrenia

Stahl et al. conducted a study on lurasidone to assess its safety and tolerability over a longer term for treating schizophrenia [19]. The secondary goal was to examine the persistence of symptom improvement. The study involved patients who had previously participated in a six-week, double-blind, placebo-controlled study of the efficacy of fixed doses of lurasidone (40 or 120 mg) or olanzapine 15 mg. These eligible patients were then given flexibly dosed lurasidone (40 to 120 mg/day) in a six-month, open-label extension study [19]. Of the 254 enrolled patients, 113 completed six months of open-label treatment. A slight decrease in mean weight and median lipid levels was observed during the open-label study. Patients previously treated with olanzapine experienced decreased weight and improved lipid levels, while patients previously treated with lurasidone or placebo experienced minimal changes [19]. There were no meaningful changes in median prolactin levels. The most reported adverse events were akathisia (13.0%) and insomnia (11.0%). Lurasidone showed persistent antipsychotic efficacy for patients who had received lurasidone, olanzapine, or placebo, with further reductions in the mean PANSS total score observed from the start of the open-label study to the final visit [19]. The results showed that flexibly dosed lurasidone (40 to 120 mg/day) was generally safe, well-tolerated, and effective over six months in patients who had completed the initial six-week, double-blind study [19].

Treatment in Asian Populations

The study by Higuchi et al. aimed to assess the effectiveness and safety of lurasidone in treating schizophrenia in Asian populations [20]. Participants from Japan, South Korea, Malaysia, and Taiwan with schizophrenia were randomly divided into groups receiving 40 or 80 mg/day of lurasidone or a placebo over six weeks in a double-blind trial [20]. The primary efficacy indicator was the change in the PANSS total score from the start to week six [20]. The analysis showed that the difference in PANSS score for lurasidone 40 and 80 mg/day compared to placebo in the population was -4.8 and -4.2, respectively. The most reported adverse events among those receiving lurasidone 40 and 80 mg/day were akathisia, somnolence, and vomiting [20]. Only a small proportion of patients experienced clinically significant weight gain (≥7%): 5.3% for lurasidone 40 mg/day, 1.3% for 80 mg/day, and 1.4% for placebo. There were no significant changes in metabolic parameters or prolactin levels in any group. The drug was well tolerated with little impact on weight and metabolic parameters [20].

Iyo et al. also sought to determine the effectiveness of lurasidone in treating acute schizophrenia in Japan and other countries [21]. The subjects, aged between 18 and 74 years and diagnosed with schizophrenia, were randomly assigned to either receive lurasidone 40 mg/day or a placebo. The primary outcome measure was the change in the PANSS total score from baseline at week six. Other efficacy evaluations included the CGI-S. Safety was assessed by monitoring adverse events, laboratory results, and electrocardiogram parameters [21]. A total of 483 subjects participated in the study. The results showed that the mean change from baseline in PANSS total scores at week six was -19.3 in the lurasidone group and -12.7 in the placebo group [21]. Changes in CGI-S scores at week six were -1.0 for Lurasidone and -0.7 for placebo. During the six-week double-blind period, the overall discontinuation rate was 19.4% for lurasidone and 25.4% for placebo, and discontinuation due to adverse events was 5.7% for lurasidone and 6.4% for placebo. The most common adverse events that occurred in more than 2% of lurasidone patients and at least twice the rate of the placebo group were akathisia (4.0%), dizziness (2.8%), somnolence (2.8%), abdominal discomfort (2.0%), and asthenia (2.0%) [21]. No significant changes in body weight or metabolic parameters were observed. Lurasidone 40 mg once daily effectively treated acute schizophrenia and was generally safe and well-tolerated in this study [21]. Table 1 summarizes these findings.

**Table 1:** Studies investigating the treatment of schizophrenia with lurasidone BPRS: Brief psychiatric rating scale, PANSS: Positive and negative syndrome scale, CGI-S: Clinical global impression-severity scale, XR: Extended-release

Reference	Number of participants	Length of study	Type of Study	Endpoint measures	Treatment arms	Results	Side effects
Nakamura et al., 2009 [17]	180	Six weeks	Double-blind trial	BPRS	Placebo (n:90), lurasidone 80 mg (n:90)	Lurasidone significantly improved BPRS scores. Lurasidone improved all secondary measures.	No significant side effects were identified with lurasidone
Loebel et al., 2013 [18]	486	Six weeks	Double-blind trial	PANSS and CGI-S	Lurasidone 80 mg (n:125), lurasidone 160 mg (n:121), quetiapine XR 600 mg (n:119), placebo (n:121)	Lurasidone improved PANSS and CGI-S with similar efficacy to quetiapine. Lurasidone had the same metabolic effects as the placebo.	No significant side effects were identified with lurasidone in this study
Higuchi et al., 2019 [19]	457	Six weeks	Double-blind trial	PANSS	Lurasidone 40 mg (n:150), lurasidone 80 mg (n:155), placebo (n:152)	Both 40 mg and 80 mg treatments with lurasidone showed significant improvement compared to the placebo group.	The most typical side effects of lurasidone were akathisia, somnolence, and vomiting
Stahl et al., 2013 [19]	254	Six weeks	Open-label extension study	PANSS	Of the 254 participants, 113 patients received lurasidone 80 mg treatment for the duration of the study.	Patients who switched from olanzapine to lurasidone experienced improved lipid labs. Patients originally on lurasidone or a placebo in the six-week trial did not experience any metabolic changes. Lurasidone decreased PANSS score in an open-label study.	Lurasidone’s most common side effect was akathisia
Iyo et al., 2021 [21]	483	Six weeks	Double-blind trial	PANSS and CGI-S	Lurasidone 40 mg (n:247), placebo (n:236)	Statistically significant improvement in PANSS and CGI-S in the lurasidone group compared to placebo. Lurasidone and placebo had similar discontinuation rates to intolerance.	The most common side effect of lurasidone was akathisia

Lurasidone in bipolar depression

Major Depressive Episodes

The study conducted by Mclyntyre et al. aimed to examine the effectiveness of lurasidone in treating major depressive episodes associated with bipolar I disorder [22]. Patients with a Montgomery-Asberg depression rating scale (MADRS) score of 20 or above and a Young mania rating scale (YMRS) score of 12 or below were randomly assigned to six weeks of treatment with either lurasidone 20 to 60 mg, lurasidone 80 to 120 mg, or placebo. Mixed features, defined as a YMRS score of four or higher, were present in 56% of patients at baseline [22]. The results showed that treatment with lurasidone was associated with significantly more significant reductions in MADRS scores in the group with mixed features and the group without mixed features compared to the placebo [22]. There was no increased risk of treatment-emergent mania observed in either group. This post hoc analysis concluded that lurasidone was efficacious in treating patients with bipolar depression who presented with mixed features [22].

The patients in the study by Loebel et al. were randomly divided into three groups, one receiving 20 to 60 mg of Lurasidone per day, another receiving 80 to 120 mg of lurasidone per day, and the last receiving a placebo in a double-blind fashion for six weeks [23]. The primary evaluation criteria were changes in the MADRS and the CGI scale for use in bipolar illness (CGI-BP) depression severity score, measured at the start of the study and again at week six [23]. The results showed that lurasidone treatment significantly improved the MADRS scores for the 20 to 60 mg/day group and the 80 to 120 mg/day group compared to the placebo group. The reduction in depression severity scores measured by the CGI-BP was also significantly more significant for both lurasidone groups than the placebo group [23]. Additionally, the patients taking lurasidone reported significantly improved anxiety symptoms, quality of life, and functional impairment compared to the placebo group. The discontinuation rates due to adverse events were comparable between the lurasidone groups (6.6% for the 20 to 60 mg/day group, 5.9% for the 80 to 120 mg/day group), and the placebo group (6.5%). The most common side effects of lurasidone were nausea, headache, akathisia, and drowsiness. Minimal changes in weight, lipid levels, or glycemic control were observed in patients taking lurasidone [16]. Lurasidone taken in the dose range of 20 to 120 mg/day was found to reduce depressive symptoms in patients with bipolar I depression significantly. Lurasidone was well tolerated, with little impact on weight or metabolic parameters [23].

Children and Adolescents

The efficacy and safety of lurasidone in treating bipolar depression in children and adolescents were evaluated in a study by DelBello et al. [2]. Patients between the ages of 10 to 17 with a diagnosis of bipolar I depression were randomly assigned to receive six weeks of treatment with lurasidone at a flexible dose of 20 to 80 mg/day or a placebo. The main measure of success was the change in the children's depression rating scale-revised (CDRS-R) total score from baseline to week six [2]. Results showed that lurasidone was associated with a statistically significant improvement in CDRS-R total score compared to placebo at week six. In addition, lurasidone also showed improvement in other secondary measures such as CGI-BP severity depression score, anxiety, quality of life, and global functioning. The completion rate for the study was 92% for the lurasidone group and 89.7% for the placebo group, with similar rates of discontinuation due to adverse events for both groups [2]. The most common side effects reported for lurasidone were nausea and somnolence. Lurasidone has minimal impact on weight and metabolic parameters [2]. 

Lurasidone in Combination With Lithium or Valproate

Suppes et al. sought to assess the effectiveness of lurasidone when used in combination with either lithium or valproate for treating bipolar I depression [24]. Participants were randomly assigned to receive lurasidone or a placebo for six weeks while continuing their background treatment with lithium or valproate. All patients had been receiving lithium or valproate treatment for at least four weeks before the start of the study. The results showed that lurasidone treatment resulted in modest, non-significant improvement compared to placebo at the six-week endpoint for the MADRS and CGI-BP scores. However, significant improvement was observed from weeks two to five for the MADRS and weeks three to five for the CGI-BP score [24]. The most common side effects reported for lurasidone were akathisia, somnolence, and extrapyramidal effects. The study suggests that lurasidone as an adjunctive treatment with lithium or valproate can improve depression symptoms from weeks two to five. Still, the effect was insignificant at the primary six-week endpoint [24].

Loebel et al. performed a similar study to Suppes et al. but had different results. In this study, one group was randomly assigned to receive six weeks of double-blind treatment with lurasidone in addition to their ongoing therapy with lithium or valproate. In contrast, the other group was assigned to receive a placebo. The results showed that lurasidone treatment significantly reduced the mean MADRS total score at week six compared to the placebo group [23]. Additionally, lurasidone treatment resulted in substantially more significant reductions in CGI-BP depression severity scores compared to placebo and significant improvements in anxiety symptoms, patient-reported quality of life, and functional impairment. The discontinuation rates due to adverse events were 6.0% and 7.9% for the lurasidone and placebo groups, respectively [23]. The most reported adverse events for lurasidone were nausea, somnolence, tremor, akathisia, and insomnia. There were minimal changes in weight, lipids, and measures of glycemic control observed during lurasidone treatment. Overall, the results sugges that in patients with bipolar I depression, lurasidone as an adjunctive treatment with lithium or valproate significantly improved depressive symptoms and was generally well tolerated [23]. Table 2 summarizes these findings. 

**Table 2:** Studies investigating the treatment of bipolar depression with lurasidone MADRS: Montgomery-Asberg depression rating scale, CDRS-R: Children's depression rating scale-revised, CGI-BP: Clinical global impressions scale for use in bipolar illness

Reference	Number of participants	Length of study	Type of study	Endpoint measure	Treatment arms	Results	Side effects
Mclntyre et al., 2015 [22]	485	Six weeks	Double-blind trial	MADRS	Bipolar depression with mixed features + lurasidone with fixed flexible dose ranges (n:182); bipolar depression without mixed features + lurasidone (n:90); bipolar depression with mixed features + placebo (n:141); bipolar depression with mixed features + lurasidone with fixed flexible dose ranges (n:72)	Lurasidone was associated with a significantly more significant reduction in MADRS scores in both mixed and without mixed features of bipolar depression.	No significant side effects were reported
DelBello et al., 2017 [2]	350	Six week	Double-blind trial	CDRS-R	Placebo (n:175), lurasidone 20 or 40 mg- (n:175)	Improved CDRS-R scores in the lurasidone group compared to placebo -Lurasidone improved secondary measures –Similar rates of adverse effects between both groups.	Lurasidone has minimal metabolic side effects.
Loebel et al., 2014 [23]	505	Six weeks	Double-blind trial	MADRS and CGI-BP	Lurasidone 20 to 60 mg (n:166), lurasidone 100 to 120 mg (n:169), placebo (n:270)	Lurasidone treatment groups had improved MADRS and CGI-BP scores. Discontinuation due to adverse effects was similar between both groups.	The most typical side effects of lurasidone were nausea, headache, akathisia, and drowsiness. Lurasidone had minimal metabolic side effects.
Suppes et al., 2016 [23]	356	Six weeks	Double-blind trial	MADRS and CGI-BP	Lurasidone flexible dose ranges (n:178), placebo (n:178)	Lurasidone did not show statistically significant improvement at the end of six weeks compared to the placebo. The lurasidone-treated group showed a significant improvement compared to controls statistically during weeks two to five.	The most common side effects reported for lurasidone were akathisia, somnolence, and extrapyramidal effects.
Loebel et al., 2014 [24]	348	Six weeks	Double-blind trial	MADRS and CGI-BP	Lurasidone 20 to 120 mg (n:183), placebo (n:165)	Lurasidone showed significant improvement in MADRS and CGI-BP scores at the end of the trial compared to the placebo group. Discontinuation due to adverse effects was similar between both groups. The most typical side effects of lurasidone were nausea, headache, akathisia, and drowsiness. Lurasidone had minimal metabolic side effects.	Adverse events for lurasidone were nausea, somnolence, tremor, akathisia, and insomnia.

Discussion

Schizophrenia is a complex and chronic mental disorder with limited treatment options. The studies we reviewed found that lurasidone is a safe and effective treatment option for individuals with schizophrenia. The studies measured efficacy through various scales including the BPRS and the PANSS. Lurasidone was found to significantly improve symptoms compared to placebo in these trials with improvement seen in all secondary measures including the PANSS total score and PANSS positive, negative, and general psychopathology subscales. The drug was well tolerated and did not cause many adverse effects on metabolic or electrocardiogram parameters, nor did it cause clinically significant differences in extrapyramidal symptoms compared to the placebo. The most common side effect of lurasidone in the studies analyzed was akathisia. The results were consistent across different studies and variable populations. Despite the existing evidence for the efficacy of lurasidone in treating schizophrenia, more drug trials need to be conducted to validate its effectiveness and safety further.

The review of lurasidone in treating patients with bipolar depression shows that it effectively reduces depressive symptoms in patients with bipolar I depression. The studies demonstrated significant improvements in depression scores compared to the placebo groups. Lurasidone has also been well tolerated with minimal side effects and little impact on weight or metabolic parameters. In studies evaluating the use of lurasidone in combination with lithium or valproate, the treatment had mixed results. More research must be done to fully understand the management of bipolar depression with lurasidone, including the optimal dose, length of treatment, and the best combination with other mood stabilizers such as valproate or lithium. Further studies should also assess the long-term safety and efficacy of lurasidone in treating bipolar depression and evaluate its effectiveness in different subpopulations of patients with bipolar depression.

## Conclusions

Lurasidone has shown much promise for its efficacy and safety in treating schizophrenia and bipolar depression. And its favorable pharmacological and metabolic profile makes it an attractive option. Additionally, clinical trials demonstrating the effectiveness of lurasidone in treating these disorders are very promising. This review provides insight into how lurasidone can successfully manage schizophrenia and bipolar depression. Several clinical trials showed that lurasidone is a highly safe and effective treatment option for patients living with schizophrenia. Also, it offers minimal risk for the development of metabolic syndrome. Lurasidone also demonstrates an ability to reduce depressive symptoms in those with bipolar I depression without provoking any substantial changes in weight or metabolism. In summary, lurasidone is an efficacious therapeutic option with a low-risk profile. More research is necessary to determine its optimal use and benefits for those with these mental health conditions.
